# Reliability of molecular host-identification methods for ticks: an experimental *in vitro* study with *Ixodes ricinus*

**DOI:** 10.1186/s13071-015-1043-7

**Published:** 2015-08-22

**Authors:** Elsa Léger, Xiangye Liu, Sébastien Masseglia, Valérie Noël, Gwenaël Vourc’h, Sarah Bonnet, Karen D. McCoy

**Affiliations:** MIVEGEC (UMR UM2-UM1-CNRS 5290, UR IRD 224), Centre IRD, 911 avenue Agropolis, BP 64501, 34394 Montpellier, Cedex 5 France; USC INRA Bartonella-tiques, UMR BIPAR ENVA-ANSES, 94706 Maisons-Alfort, France; Laboratory of Infection and Immunity, Xu Zhou Medical College, 221004 Xu Zhou, P.R. China; Unité Epidémiologie Animale (UR INRA 346), Centre de recherche INRA de Clermont-Ferrand / Theix, 63122 Saint Genès Champanelle, France

**Keywords:** Host identification, Hematophagous arthropods, Ixodidae, Molecular methods, Detection biases, Vector-borne diseases

## Abstract

**Background:**

Reliable information on host use by arthropod vectors is required to study pathogen transmission ecology and to predict disease risk. Direct observation of host use is often difficult or impossible and indirect methods are therefore necessary. However, the reliability of currently available methods to identify the last host of blood-feeding arthropods has not been evaluated, and may be particularly problematic for ticks because host blood has been digested at capture. Biases in host detection may lead to erroneous conclusions on both vector ecology and pathogen circulation.

**Methods:**

Here, we experimentally tested for biases in host detection using the generalist three-host tick *Ixodes ricinus* as a model system. We fed ticks using an artificial feeding system and amplified blood meal traces post-moult (i.e., in the succeeding unfed life stage) via both a quantitative real-time polymerase chain reaction assay and a reverse line blotting method. We then experimentally tested for three types of biases in host detection: 1) time post-moult, 2) tick life stage and 3) host type (non-nucleated mammal blood versus nucleated avian blood), and compared these biases between the two molecular methods.

**Results:**

Our results show that all three factors can influence host detection in ticks but not necessarily in the expected way. Although host detection rates decreased with time post-moult, mammal blood tended to be more readily detected than bird blood. Tick life stage was also an important factor; detection was higher in nymphs than in adults and, in some cases, remnants from both larval and nymphal blood meals could be detected in the adult stage. These biases were similar for the two detection techniques.

**Conclusions:**

We show that different factors associated with questing ticks may influence our ability to correctly infer previous host use and that these factors may bias inferences from field-based studies. As these biases may be common to other vector-borne disease systems, their implications for our understanding of vector ecology and disease transmission require more explicit consideration.

**Electronic supplementary material:**

The online version of this article (doi:10.1186/s13071-015-1043-7) contains supplementary material, which is available to authorized users.

## Background

Vector-borne diseases are often maintained in complex transmission cycles that include numerous potential reservoir host species. To understand vector ecology and pathogen circulation, and to reliably predict disease risk, the contribution of different host types to local vector-host-pathogen interaction networks needs to be established [1–4]. Molecular techniques, such as group-specific polymerase chain reaction (PCR), restriction fragment length polymorphisms (RFLP), reverse line-blot hybridisation (RLBH) and DNA barcoding, are currently employed to identify host use in a wide range of blood-feeding arthropods (e.g., mosquitoes, ticks, reduviid bugs, sandflies, fleas, tsetse flies, biting midges) without the need to directly collect the vector on the host [5, 6]. The overall success in host identification is known to vary across techniques and vector systems (e.g., [7–14] and see [5, 6] for reviews), but the representativeness of the detected hosts in terms of correctly determining the way the natural systems function has not been evaluated to date. Undetected biases could result in an erroneous perception of local host use and, thus, of pathogen circulation. In the present study, we conducted a controlled experiment to identify and quantify potential biases in host detection in ticks using the well-known generalist tick *Ixodes ricinus* (Acari, Ixodidae) as a model system.

*I. ricinus* is a three-host tick, with a single compulsory blood meal during each of its three active stages: larva, nymph and adult (except for adult male). The complete cycle of this tick is rather long, taking between two and six years to complete [15] with a relatively long interval between blood meals. *I. ricinus* is the most widespread and abundant tick in western Europe, parasitising a vast range of terrestrial vertebrates including mammals, birds, and reptiles, and transmitting a wide variety of viral, bacterial and parasitic diseases of medical and veterinary importance [16], including Lyme borreliosis, the most prevalent vector-borne zoonosis in both Europe and North America [17].

Potential biases in host detection/identification could arise through several factors in species such as *I. ricinus*. First, due to their large host range, variation in detection can occur because certain host types may be more easily detected than others due to differences in the amount of ingested DNA (e.g., nucleated versus non-nucleated host red blood cells) [5, 8, 18, 19] or to the presence of amplification inhibitors [20–22]. Detection may also vary with the tick life stage. Indeed, hard ticks are frequently sampled off-host during the questing phase, when the blood meal from the preceding life stage has been completely assimulated. As blood digestion is intracellular, with digestive cells of the midgut phagocytising the blood using specialised structures called endosomes, small amounts of undigested blood from the previous blood meal may be stored [23, 24]. This allows DNA-based detection of vertebrate blood meals for a greater period of time than for other hematophagous arthropods, but also means that whereas questing nymphal ticks should only carry host DNA from the larval blood meal, questing adult ticks may carry trace DNA from both larval and nymphal blood meals [8, 19, 25, 26]. Finally, previous studies have also suggested that the time since the last blood meal is an important factor to consider in the ability to identify host use because host DNA remnants may continue to degrade over time [12, 18, 26, 27].

**Fig. 1:**
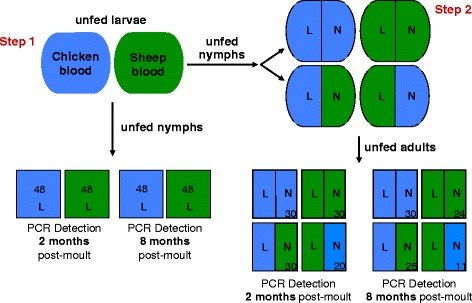
Experimental design for testing host detection probabilities in ticks. In step 1, unfed larvae were fed on sheep or chicken blood. Engorged larvae were then maintained under standard laboratory conditions until the moult. One half of the newly moulted nymphs were kept for host detection analysis. In step 2, the other half of unfed nymphs from step 1 were again fed using the artificial feeding system with four treatment types: nymphs fed as larvae on one host blood type were either fed again on the same blood type (blood meal treatments “sheep-sheep” and “chicken-chicken”) or on a different blood type (blood meal treatments “sheep-chicken” and “chicken-sheep”). Fully-engorged nymphs that detached from the membrane were kept under standard laboratory conditions until the moult into the adult life stage. The sample sizes of ticks analysed for host use are indicated for each treatment. Blue represents chicken blood meal and green sheep blood meal. L = larval blood meal, > N = nymphal blood meal

Using a full cross-feeding design (Fig. 1) combined with an artificial feeding system [28–30] and two different molecular methods for amplifying host blood meal traces (quantitative real-time polymerase chain reaction (qPCR) and reverse line blot hybridisation (RLBH) methods), we experimentally tested for biases in host detection in *I. ricinus*. In terms of host type, we used two blood sources (bird and mammal) and hypothesized that avian blood would have higher overall detection rates and thresholds than mammalian blood due to differences in the amount of DNA ingested by feeding ticks; birds have nucleated red-blood cells whereas mammals do not. Although host DNA is also present in white blood cells, lysed subcutaneous tissue and skin cells, differences in the quantity of DNA in red blood cells (nucleated versus non-nucleated) can be substantial (see Methods) and we therefore focused on this aspect. We also predicted that host detection would be higher in adult ticks than in nymphal ticks due to larger blood meal size in the previous life stages. Finally, we expected that that detection would decrease with time post-moult due to DNA degradation in the midgut. We discuss the significance of our results for understanding the reliability of host molecular detection methods and the consequences of potential biases for our understanding of vector-borne diseases.

## Methods

### Ticks

All experiments were performed with *I. ricinus* ticks from a pathogen-free laboratory colony, reared at 22 °C with 80–90 % relative humidity and with a 12 h light/dark cycle [28–30]. This colony has been maintained over several generations in the CRBM (Centre de Recherches Biomédicales) in Maisons-Alfort, but is regularly supplemented with adult ticks from the wild. Field collected females are engorged on rabbits and maintained in standard laboratory conditions until egg-laying. After egg-laying is complete, the DNA from these females are extracted and tested for the presence of pathogens. Only progeny from uninfected mothers are added to the colony [29]. Ticks of the colony are typically fed artificially on sheep blood in the larval stage and directly on laboratory rabbits in the nymphal and adult life stages.

### Experimental design

In order to test for biases using indirect host detection methods, our experimental design included two major feeding steps, one at the larval stage and one at the succeeding nymphal stage, and four experimental groups according to the source of host blood (bird/bird, mammal/mammal, bird/mammal, mammal/bird). At each step, a random sample of unfed ticks from each group was tested for host DNA traces at both two and eight months post-moult to determine the effect of time on detection. The details of the experimental design and sample sizes are presented in Fig. 1.

### Tick engorgement, maintenance and DNA extraction

The artificial feeding system employed has been previously described [28, 30] and we therefore only provide supplementary information here. Two types of skin membranes were used on the artificial feeders, either gerbil (*Meriones unguiculatus*) or rabbit (*Oryctolagus cuniculus*). The blood supply for the artificial feeders came from two sources: sheep (*Ovis aries*) blood purchased from BioMerieux Laboratories (France), and chicken (*Gallus gallus*) blood taken directly from living animals maintained on site. As expected, substantial differences in the amount of DNA present in each blood meal source were apparent: 1.3 mg DNA/g of chicken blood and 0.003 mg DNA/g of sheep blood, quantified using a Tecan NanoQuant Plate Infinite 200. After moulting, unfed nymphal and adult ticks from the different experimental groups were maintained separately under the standard laboratory conditions mentioned above. After the planned post-moult period, ticks were placed directly at −80 °C until DNA extraction.

Prior to DNA extraction, ticks were washed twice in distilled water to eliminate impurities. The whole body of the tick was then ground using a sterile piston and scissors. DNA was extracted using the DNeasy® Blood & Tissue Kit (QIAGEN, Hilden, Germany) following manufacturer’s instructions. DNA extracts were then stored at −20 °C.

### Real-time qPCR assay

We developed a quantitative real-time polymerase chain reaction (qPCR) assay based on the amplification of a partial fragment of the 12S gene in an attempt to simultaneously detect and quantify host DNA. Sheep and chicken 12S rDNA sequences were downloaded from GenBank [AF010406; AY235571, AP003580.1] and aligned using BioEdit [31]. Specific forward and reverse primers were designed using Primer3Plus [32] (Table 1). qPCR assays were performed using a LightCycler®480 PCR instrument (Roche Diagnostics, Mannheim, Germany). Both DNA concentration and the amplification program used were optimised for the DNA templates. Reactions were performed in 10 μL reaction mixtures containing 2x LightCycler®480 SYBR®Green I Master, 10 μM of each primer and 1 μL of tick DNA extract (diluted to approximately 10 ng/μL). The amplification program consisted of an initial 10 min denaturation step at 95 °C, followed by 50 cycles with 10 s of denaturation at 95 °C and 1 min of annealing and extension at 64 °C. Fluorescence was measured at the end of each extension step. After amplification, a melting curve was acquired by heating the product at 20 °C/s to 95 °C, cooling it at 20 °C/s to 60 °C, keeping it at 60 °C for 20 s, and then slowly reheating it at 0.1 °C/s to 95 °C. During this stage, fluorescence was measured through the slow reheating phase.

**Table 1:** Primer sequences used for the qPCR assay and probes and PNA sequences used for the Reverse line blotting method in this study

Name	Target organism	Position	Sequence
12Sov	Sheep (*Ovis aries*)	Forward	CCAGCCTTCCTGTTAACTTTCAATAGACT
		Reverse	TTTAGTCCTGTGTGATTCGAAGGGCG
12Sgg	Chicken (*Gallus gallus*)	Forward	CTCGCTAATAAGACAGGTCAAGGTA
		Reverse	TAGGGGGTATGATCTCACTTTACTG
Chicken probe	Chicken (*Gallus gallus*)	-	Amino-ACCTCCCATCACACATGT
Sheep probe	Sheep (*Ovis aries*)	-	Amino-AAATAATTATAAAAACAAAATTATTC
PNA clamp	Human (*Homo sapiens*)	Reverse	H-GTGTTCTGGCGAGCAGTT-NH2

PCR efficiency was measured for each target by generating standard curves with DNA isolated from the whole blood of each host type, diluted 10 fold, 4 times, from a starting concentration of 5 ng/μL at 1x. We verified that PCR efficiency and sensitivity was not different between chicken and sheep target. A threshold cycle (Ct) was determined by the software LightCycler®480 1.5.0 based on the amplification curves of the positive individuals; the lower the Ct, the higher the amount of starting template material. Samples with a Ct greater than 45 were considered to have no identifiable blood meal. The specificity of qPCR products was confirmed by a melting curve analysis (Tm) and control profiles. We used DNA extracts from sheep, chicken, gerbil and rabbit blood as positive controls and sterilised water was used as a negative control. All assays were repeated three times in independent runs.

### Reverse line blotting method

#### Probe design and peptide nucleic acid clamp

We used the primers (12S-6F and B12S-9R) and the reverse line blot (RLB) probes for mammals, birds, chicken, sheep and humans as previously designed [25]. In addition, new specific probes were designed for chicken and sheep (Table 1) using 12S rDNA sequences downloaded from GenBank: 96 from birds, 152 from mammals and 12 from lizards. Sequences were aligned using Muscle multi-alignment software [33].

As human DNA can be an important contaminant when using RLB methods and can compete with target DNA [19, 25], we designed a Peptide Nucleic Acid (PNA) clamp [34] to block human 12S mitochondrial gene amplification. The clamp was designed using the alignment of 260 vertebrate sequences of rDNA 12S and targeted 18 nucleotides located on the ~145 bp 12S rDNA amplified fragment. The human-targeted sequence presented a minimum of three mismatches with the other aligned sequences to guarantee specificity of the PNA clamp. The clamp melting temperature (Tm) is 12 °C higher than the primer melting temperatures [35–37]. A special 30 s annealing step at 60 °C allowed the formation of the PNA/DNA complex on human 12 s rDNA before primer annealing. This blocked the extension of the reverse biotin labelled primer upstream from the hybridisation site of *Homo sapiens* RLB probe.

#### PCR amplification

The amplification of the ~145 bp fragment of vertebrate 12S rDNA was based on a protocol adapted from [25]. Reactions were performed in 50 μL reaction mixtures containing 0.5 μM of the human PNA Clamp, 0.8 μM of 12S-6F and B12S-9R primers, 1.25U of Taq DNA Polymerase with 1x Q-Solution (QIAGEN) and 10 μL of DNA extract from ticks. PCRs were performed in a C1000 thermocycler (Bio-Rad, Hercules, California, USA). The PCR program consisted of an initial denaturation step of 3 min at 94 °C before the nine first cycles (20 s at 94 °C for denaturation; 30 s at 60 °C for human PNA clamp annealing; 30 s at 60 °C, with temperatures dropping by 1 °C at each cycle until 52 °C for primer annealing; 30 s at 72 °C for extension). These touchdown cycles were followed by 40 cycles at constant temperatures (20 s at 94 °C; 30 s at 60 °C; 30 s at 52 °C; 30 s at 72 °C). Amplified fragments were subsequently analysed using reverse line blotting.

#### Reverse line blot hybridisation

Membranes were prepared with one probe from each target. PCR products were incubated with every probe by reverse line blotting on both membranes as described in [25]. The *H. sapiens* probe was used to verify PNA clamp efficiency. To reveal hybridized biotin labelled PCR products, membranes were incubated for 30 min at 42 °C with a 10,000× diluted Streptavidin IR-Dye 800 (LI-COR, Bad Homburg, Germany). Membranes were then washed twice for 10 min at 42 °C in 100 mL of a 2X SSPE/0.5% SDS solution and twice for 5 min at ambient temperature in 100 mL of a 2X SSPE solution. Then, the IR Dye of Biotin-Streptavidin complex was scanned at 800 nm with an Odyssey® infrared imaging system (LI-COR). Assays on nymphs 2 months post-moult were repeated three times in independent runs. Only one run was performed on the other ticks.

### Tick measurements

As tick size can vary with the quality and quantity of the blood meal [38–40], we measured the length and width of a sub-sample of ticks from each experimental group to provide us with information on the relative quality of different host blood types. As measurements were made on ticks that were not used in host detection, adults of the group “sheep/chicken” were not measured due to low sample sizes (see Additional file 1: Table S1).

All measurements were made using a binocular microscope and the software Leica S.A.S.

### Data analysis

We considered three variables associated with host detection: 1) the detection threshold obtained with the qPCR method, a continuous variable defined by the Ct cycle at which a positive amplification was found and which reflects the relative quantity of host DNA present; the lower the Ct, the higher the amount of DNA template, 2) the detection rate, that is, the proportion of positive samples found for each experimental group with both RLBH and qPCR techniques and 3) the tick size and how it varied according to host blood type.

We used generalised linear models (GLM) to analyse data. Full models were simplified using a stepwise backward procedure which consisted in sequentially eliminating non-significant terms and interactions (at the 0.05 level) to obtain a minimal model [41, 42]. All statistical analyses were carried out using the lme4 package implemented in the R v3.0.1 statistical software (R Foundation for Statistical Computing, Vienna, Austria).

#### Detection threshold (Ct)

We used all positive ticks obtained with the qPCR method to analyse the detection threshold for each experimental group. As these data did not follow a normal distribution, we applied a boxcox transformation (γ = −7) to obtain normality [41]. We used “host type”, “time post-moult” and “tick life stage” as fixed explanatory variables and determined their significance using a *χ*^2^ test.

#### Detection rate

Overall detection rate, corresponding to the sensitivity of the method, was measured for each detection technique. Detection rates of qPCR and RLBH techniques (for one run only) were compared using Chi-Squared Contingency test. In addition, repeatability in detection within and between techniques was calculated based on results from individual tick extracts.

Due to differences in the number of blood meals and blood meal size, we analysed ticks from Step 1 (nymphs) and Step 2 (adults) separately (Fig. 1). Within Step 2, we separated adult ticks originated from larvae and nymphs fed on the same host type (Group 1) from those issued from larvae and nymphs fed on different hosts (Group 2). As adult ticks were only analysed once with the RLBH method, we used only the data obtained from the first run of the qPCR assays for comparing the detection rates of the two methods. In the GLM, “tick” was included as a random nested factor and we considered a binomial distribution for the response variable. In nymphs and in Group 1 adults, “method”, “host type” and “time post-moult” were used as explanatory variables for host detection rate. In Group 2, “method”, “host type”, “blood meal life stage” (larval or nymphal) and “time post-moult” were used as explanatory variables for host detection rate. The significance of these explanatory variables was inferred from likelihood ratio tests (LRT).

#### Tick size

As for detection rate analyses, we analysed ticks from Step 1 (nymphs) and Step 2 (adults) separately (Fig. 1). We used “host type” and “blood meal life stage” (only for adults) as explanatory variables and determined their significance using F tests.

## Results

### Detection threshold

**Fig. 2:**
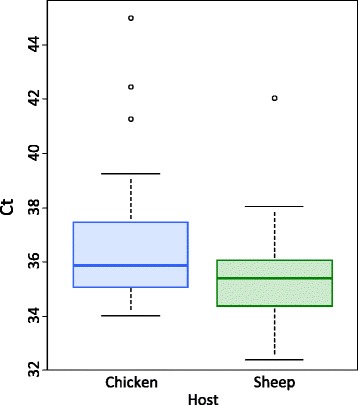
Overall threshold number of cycles (Ct) for the detection of chicken (*N* = 33) and sheep (*N* = 40) blood in moulted ticks

No differences were observed in the detection threshold at 2 months and 8 months post-moult (χ^2^_1_ = 3.13e-25, *p* = 0.41) nor between nymphs and adults (χ^2^_1_ = 1.28e-25, *p* = 0.6). Only the origin of the blood meal had a significant effect on the detection threshold (χ^2^_1_ = 3.89e-24, *p* = 0.004) (Additional file 1: Table S2); the detection threshold was lower in ticks when fed on sheep-blood compared to those fed on chicken-blood (sheep: Ct = 35.33 ± 1.71, chicken: Ct = 37.28 ± 3.43) (Fig. 2). These results suggest that, in contrast to predictions, more template DNA was present in sheep-fed ticks post-moult than in bird-fed ticks and that this remnant DNA remained stable over at least an 8-month period.

### Detection rate

We achieved an overall detection rate of 20 % for nymphs and 12 % for adults with qPCR and 22 % for nymphs and 15 % for adults in RLBH. Although sensitivity was significantly higher for the RLBH method (*χ*^2^ = 4.03, *p* = 0.04), the repeatability between techniques was reasonably high. Indeed, repeatability was lower among runs within techniques, 77.6 % for qPCR and 73.4 % for RLBH than between techniques (82.5 %).

**Fig. 3:**
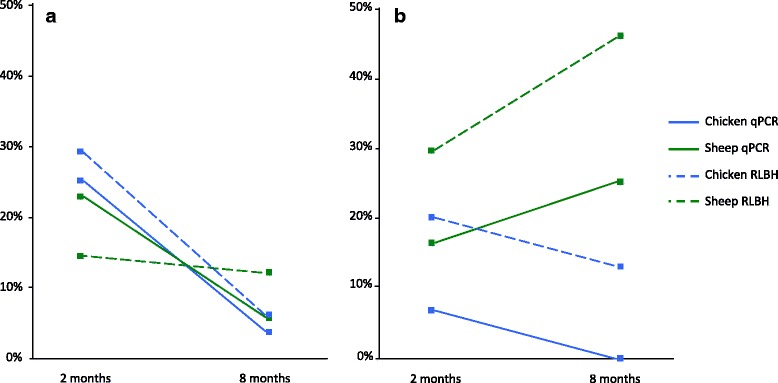
Host detection rates at 2 months and 8 months post-moult for both qPCR and RLBH methods for (**a**) nymphal ticks (48 ticks were tested in each group) and (**b**) adult ticks which fed on the same host type at larval and nymphal stages (30 ticks were tested in each group except for sheep ticks 8 months post-moult for which only 24 ticks were analysed). For ease of interpretation, we have linked treatment groups at the two time intervals

The detection rate in nymphal ticks was strongly dependant on time post-moult (χ^2^_1_ = 15.88, *p* < 0.0001), decreasing over the 6 month interval, but neither host type (χ^2^_1_ = 0.31, *p* = 0.58), nor detection method (χ^2^_1_ = 0.10, *p* = 0.75) had significant effects (Fig. 3a, Additional file 1: Table S2).

In Group 1 adults (in which larval and nymphal stages fed on the same host type), there was a significant interaction between host type and time post-moult (χ^2^_1_ = 4.48, *p* = 0.03); detection rate increased in sheep-fed ticks whereas it decreased in chicken-fed ticks. There was also a difference between qPCR and RLBH detection rates at this life stage (χ^2^_1_ = 10.2, *p* = 0.001), where rates were higher using the RLBH method (Fig. 3b, Additional file 1: Table S2).

In Group 2 adults (in which larval and nymphal ticks fed on different host types), there was a significant interaction between time post-moult and stage (χ^2^_1_ = 3.94, *p* = 0.04) and between time-post-moult and host type (χ^2^_1_ = 3.94, *p* = 0.04) (Table 2, Additional file 1: Table S2). However, these results should be taken with caution considering the overall low number of successful detections (Table 2).

**Table 2:** Host detection rate (%) in adult ticks per method, life stage at which blood meal was taken on each host type and time post-moult

Time post-moult	qPCR		RLBH		N
	Larvae	Nymph	Larvae	Nymph	
2 months	10.00	**5.00**	5.00	**10.00**	20
	**3.33**	3.33	**16.67**	3.33	30
8 months	0	**9.09**	9.09	**0**	11
	**0**	20.00	**4.00**	12.00	25

N refers to the number of ticks tested. Bold numbers indicate ticks fed on chicken blood and unbold numbers ticks fed on sheep blood (See Fig. 1 for details)

### Tick size

As the length and width of ticks were strongly correlated (Pearson’s correlation; r = 0.98, *p* < 0.0001), we used only length to compare tick size post-moult. In unfed nymphs, size was only influenced by host type used for the larval blood meal (F_87,1_ = 10.1, *p* = 0.002) (Additional file 1: Table S2); sheep blood-engorged ticks were larger than chicken blood-engorged ticks (chicken ticks: length = 1.09 ± 0.07, width = 0.706 ± 0.05; sheep ticks: length = 1.13 ± 0.06, width = 0.783 ± 0.05). However, this difference was not evident in adults (F_133,2_ = 1.38, *p* = 0.25) (Additional file 1: Table S2).

## Discussion

Numerous studies on vector feeding behaviour have been carried out to better understand vector ecology and to identify potential vertebrate reservoirs of vector-borne pathogens (e.g., [8, 11, 27, 43–47]). Ticks are unique among blood-sucking arthropods as their blood meal typically occurs several months before capture and is largely digested when detection is performed. This trace amount of host DNA, combined with a large host spectrum, makes host identification particularly challenging for certain tick species. In this context, the aim of the present study was to investigate the potential biases associated with indirect host identification methods used for ticks and whether these biases require explicit consideration when making inferences about tick ecology and pathogen transmission cycles. Our results suggest that several factors may indeed affect our image of host use and that these biases are present across molecular techniques (here, qPCR and RLBH).

Molecular host detection methods in questing ticks have a general success rate of approximately 50% [18, 19, 25–27, 47]. In our study, overall detection rates were lower than in field-collected ticks, varying between 12 and 20% and 15 and 22% for qPCR and RLBH methods respectively. This low sensitivity could be due to the fact that ticks were engorged using an artificial feeding system. In addition to abiotic conditions that change in the laboratory, ticks may show host specificity [48–51] or preferences for particular physical and physiological properties of the host that favour feeding under natural conditions. Indeed, attempts to rear different tick species under laboratory conditions have frequently met with limited success [29, 30, 51, 52]. The artificial feeding system we used may also have reduced host detection in another way. In our experiment, the skin used in the feeding apparatus came from a different host type (gerbil or rabbit) than the blood. If remnant host DNA in ticks comes from a mix of digested tissues, skin, white blood and red blood cells rather than from blood only, the low detection rates found in our study might be expected. Indeed, controls during host detection assays showed that both gerbil and rabbit DNA was highly detectable in ticks (results not shown). Despite these potential limitations, the parameters we measured in our study (detection threshold, detection rate and tick size) were obtained under the same artificial feeding conditions, such that comparisons between techniques, hosts, life stages and time are valid, even if overall detection is lower than might be expected under natural conditions.

Detection thresholds differed significantly among host types, but not in the predicted direction. Among positive individuals, amplifications started earlier in sheep-fed ticks compared to chicken-fed ticks suggesting a higher quantity of host DNA when ticks fed on sheep during the previous blood meal. Likewise, sheep blood tended to have higher detection rates than chicken blood, although the difference was not significant. There are several alternative explanations for these unexpected results. Here, nymphs that fed on sheep blood as larvae were generally larger than nymphs that fed on chicken blood. This could suggest that a greater amount of sheep blood (and therefore sheep DNA) was imbibed, on average, compared to chicken-fed ticks during engorgement. However, a recent study using the same basic protocol found that post-engorgement weights are similar for *I. ricinus* ticks that have fed on avian and mammalian blood sources [30]. We know from previous work that host blood quality may influence the size of the vector [51, 53–55] but also the success of DNA amplifications [56, 57]. The variation we observed could thus be due to differences in host blood characteristics or to host immune responses which reduce blood meal quality [58, 59]; this latter explanation could be particularly important within the framework of the present study because fresh chicken blood came directly from living animals rather than via a pharmaceutical company, and therefore may have contained active immune components that were no longer present in the sheep blood. Differences in host quality could also be linked to host specificity in the vector population; the vector being locally adapted to exploit a preferred host [48, 49, 51, 57, 60]. In our case, the laboratory-reared ticks typically feed on mammalian food sources and may therefore be better adapted to exploit these hosts. Finally, both detection techniques used here rely on the amplification of host DNA in the tick DNA extract. During haemoglobin digestion there is a concentration of residual heme molecules [61] that can act as a PCR inhibitor [20–22]. DNA amplification could therefore vary among host types due to differences in the presence of these inhibitors. Although, we cannot differentiate among these alternative explanations with the present data, our results demonstrate that host type can significantly modify host detection parameters.

Detection rates were lower in adult ticks compared to nymphal ticks. This was surprising, as we expected that the larger blood meal taken in the nymphal life stage would increase detection probability in questing adult ticks. However, given the relative size difference between nymphal and adult life stages, and the difference in the physiological demands of nymphal and adult moults, it may be that the relative amount of host remnant DNA has nothing to do with blood meal size *per se*. In accordance with our results, Scott et al. (2012) also report a slightly higher detection rate in *Amblyomma americanum* nymphs compared to adults, without discussing this in more detail [19]. In a few cases, we were able to detect blood remnants from both larval and nymphal blood meals in the same unengorged adult tick. This observation supports previous findings of mixed blood meals in wild adult ticks, results that are frequently (and potentially erroneously) attributed to contaminations [8, 19, 25, 26].

In ticks, DNA-based detection of vertebrate blood meal is possible over long periods of time. In our study, the detection rate was globally higher two months post-moult than eight months post-moult, as expected from previous work [12, 18]. A decline in detection over time has also been found in other hematophagous vectors, but over much shorter time scales [62–64]. However, in adult ticks, sheep detection rates actually increased eight months post-moult, suggesting that previous observations could be host-dependant. Indeed, Woods et al. (2009) have shown that, in fleas, the decrease in detection over time varies among host species [46]. Morán-Cadenas et al. (2007) and Pichon et al. (2003) found seasonal variation in host blood meal detection success, but could not differentiate between temporal variation in host species exploitation and time since the last blood meal [26, 27]. Temporal changes in host detection therefore require explicit consideration when studying blood-feeding behaviour in natural populations, because these changes may or may not reflect true changes in host use. Future work aimed at determining the physiological mechanism behind variation in host detection rates is also called for in order to better understand the origin of this variation and to control for it.

Our results highlight some of the problems that can be encountered when using indirect molecular methods to identify the last host used by a vector. As the goal is to amplify degraded DNA in trace quantities, a first major issue can be contamination. This was particularly a problem for the RLBH technique that includes a wide range of host specific probes. We were able to overcome this issue for human DNA, a frequent contaminate in such studies, by using a PNA clamp. This problem is more difficult to control for other vertebrate host species when using field collected tick specimens and requires specific consideration in detection protocols. Second, although there was relatively high repeatability in individual host detections between qPCR and RLBH methods (82 %), it was somewhat lower among runs for a given technique (~75 %). Part of this variation may be due to the probability of pipetting host DNA, present in a small quantities compared to tick DNA. Host detection success in field-collected ticks could therefore be greatly improved by performing replicate assays on the same vector DNA extraction; we recommend running triplicate tests. Here, we compared two methods of host detection, but the utilisation of newer techniques, such as NGS, proteomic or isotopic analyses [1, 65–69], could prove more efficient. A comparison of these techniques would be useful for optimizing host detection probabilities and reducing detection biases.

## Conclusions

Biases in host identification can lead to erroneous conclusions on vector feeding ecology and local host use and thus can have severe consequences for understanding patterns of pathogen transmission in nature. Our study, based on an artificial laboratory system, demonstrated that different biases can alter our ability to make robust conclusions for ticks from natural populations. Host effects are particularly important to consider in future work, and notably in relation to how vector adaptation may alter our ability to detect host use. For example, as blood quality may depend on how well ticks are adapted to exploit a particular host type (e.g., ability to digest chicken blood in the present study), the use of novel hosts may be more difficult to detect than typical hosts. This issue could be particularly problematic if we want to measure the role of introduced host species on tick population dynamics and pathogen transmission. Our conclusions focus specifically on tick vectors, but potential biases also likely occur in other hematophagous vector species. The investigation of these issues in other vector systems is therefore called for and, particularly so in those systems where host feeding can be controlled under laboratory conditions.

## Additional file

Additional file 1: Table S1. Number of ticks measured in each treatment group of the experiment. **Table S2.** Description of statistical models used to analyse the influence of host type (“host”), time post-moult (“moult”), tick life stage (“stage”), bloodmeal life stage (“bloodmeal”) and method as explanatory fixed variable and tick as a random nested factor on detection threshold, detection rate and tick size. N gives the number of ticks included in each analysis. “Maximal model” gives the complete set of explanatory variables tested (and their interactions) included in the model. “Minimal model” gives the model containing only the significant variables and their interactions. (DOCX 16 kb)
