# Supplementary material for: Reliability of molecular host-identification methods for ticks: an experimental in vitro study with Ixodes ricinus
Source: Parasit Vectors. 2015 Aug 22;8:433. doi: 10.1186/s13071-015-1043-7 (PMC4546307; doi:10.1186/s13071-015-1043-7)
Supplement: Additional file 1: Table S1. — Number of ticks measured in each treatment group of the experiment. Table S2. Description of statistical models used to analyse the influence of host type (“host”), time post-moult (“moult”), tick life stage (“stage”), bloodmeal life stage (“bloodmeal”) and method as explanatory fixed variable and tick as a random nested factor on detection threshold, detection rate and tick size. N gives the number of ticks included in each analysis. “Maximal model” gives the complete set of explanatory variables tested (and their interactions) included in the model. “Minimal model” gives the model containing only the significant variables and their interactions. (DOCX 16 kb) [file 13071_2015_1043_MOESM1_ESM.docx]

**Additional File 1**

**Table S1.** Number of ticks measured in each treatment group of the experiment

| **Stage** | **Treatment** | **N** |
| --- | --- | --- |
| Nymph | chicken | 45 |
| Nymph | sheep | 43 |
| Adult | chicken-chicken | 58 |
| Adult | sheep-sheep | 31 |
| Adult | chicken-sheep | 45 |

**Table S2.** Description of statistical models used to analyse the influence of host type (“host”), time post-moult (“moult”), tick life stage (“stage”), bloodmeal life stage (“bloodmeal”) and method as explanatory fixed variable and tick as a random nested factor on detection threshold, detection rate and tick size. N gives the number of ticks included in each analysis. "Maximal model" gives the complete set of explanatory variables tested (and their interactions) included in the model. "Minimal model" gives the model containing only the significant variables and their interactions.

| **Variable of interest** | **N** | **Maximal model** | **Minimal model** |
| --- | --- | --- | --- |
| Detection threshold | 73 | host*moult*stage | host |
| Nymphal detection rate | 192 | host*moult*method+(tick) | moult+(tick) |
| Adult detection rate (group 1) | 228 | host*moult*method+(tick) | host*moult+method+(tick) |
| Adult detection rate (group 2) | 344 | host*moult*bloodmeal*method+(tick) | host*moult+moult*bloodmeal+(tick) |
| Nymphal tick size | 88 | host | host |
| Adult tick size | 134 | host* bloodmeal | 1 |
